# Barley somatic embryogenesis-an attempt to modify variation induced in tissue culture

**DOI:** 10.1186/s40709-021-00138-5

**Published:** 2021-03-16

**Authors:** Renata Orłowska

**Affiliations:** Plant Breeding & Acclimatization Institute–National Research Institute, 05-870 Błonie, Radzików, Poland

**Keywords:** Optimisation, Taguchi, metAFLP, Barley, Somatic embryogenesis

## Abstract

**Background:**

Somatic embryogenesis is a phenomenon carried out in an environment that generates abiotic stress. Thus, regenerants may differ from the source of explants at the morphological, genetic, and epigenetic levels. The DNA changes may be the outcome of induction media ingredients (i.e., copper and silver ions) and their concentrations and time of in vitro cultures.

**Results:**

This study optimised the level of copper and silver ion concentration in culture media parallel with the induction medium longevity step towards obtaining barley regenerants via somatic embryogenesis with a minimum or maximum level of tissue culture-induced differences between the donor plant and its regenerants. The optimisation process is based on tissue culture-induced variation evaluated via the metAFLP approach for regenerants derived under varying in vitro tissue culture conditions and exploited by the Taguchi method. In the optimisation and verification experiments, various copper and silver ion concentrations and the different number of days differentiated the tested trials concerning the tissue culture-induced variation level, DNA demethylation, and de novo methylation, including symmetric (CG, CHG) and asymmetric (CHH) DNA sequence contexts. Verification of optimised conditions towards obtaining regenerants with minimum and maximum variability compared to donor plants proved useful. The main changes that discriminate optimised conditions belonged to DNA demethylation events with particular stress on CHG context.

**Conclusions:**

The combination of tissue culture-induced variation evaluated for eight experimental trials and implementation of the Taguchi method allowed the optimisation of the in vitro tissue culture conditions towards the minimum and maximum differences between a source of tissue explants (donor plant) and its regenerants from somatic embryos. The tissue culture-induced variation characteristic is mostly affected by demethylation with preferences towards CHG sequence context.

## Background

Genetic or epigenetic purity of regenerants can be perturbed by e.g. chromosomal changes [1], point mutations [2], movement of transposable elements [3], or changes in methylation status of DNA [4] originating due to numerous stresses (sterilization factors [5], media components [6], light conditions [7] or humidity [8]). The extent of changes may depend on a type of explants [9], genotype [10], or time of culture on induction medium [11, 12].

Many reports pointed out media components as the factors inducing genetic [13] or epigenetic [14] changes shared among in vitro derived plant. Among media components, copper and silver ions are indispensable for plant growth and development. Copper ions at the physiological level are crucial for growth and plant survival [15]. A somewhat elevated level of copper ions in induction or regeneration media may positively influence somatic embryogenesis [16, 17] or androgenesis [18]. However, overstepping bounds of toxic doses may imbalance the chloroplast redox state altering photosynthesis [19–21], and inducing oxidative stress [22]. Another ion utilized in tissue culture media is silver [23]. The positive effect of AgNO_3_ on somatic embryogenesis, shoot growth, and direct organogenesis is well established [24]. However, at increased concentration silver ions may be toxic for plant tissues inducing reactive oxygen species [25] followed by oxidative DNA damages [26, 27]. Silver ions may also influence retrograde (plastid-to-nucleus) signaling [28], epigenetic processes [29] and activate reparation mechanisms [30].

The other factors that may affect (epi)genetic purity of regenerants are, i.e., time of in vitro induction medium [31, 32] and the presence of callus stage [33]. In contrast to direct embryogenesis [34], indirect one may induce somaclonal variation at the DNA sequence level due to callus phase [35]. Studies devoted to the mutation rate in plant tissue cultures pointed at the prevalence of transition over transversion. However, with increasing tissue culture time, the transversion rate also rises [36]. Apart from DNA mutations, changes in ploidy were observed when tissue cultures kept for a prolonged time [37].

Although rare, the influence of different factors affecting tissue culture plant regeneration can be seen at the morphological level of regenerants [10]. However, the lack of such changes does not mean they are not present at the genetic or DNA methylation levels [38, 39]. DNA-based marker techniques, including Methylation Sensitive Amplification Polymorphism (MSAP) [40, 41] or methylation-sensitive Amplified Fragment Length Polymorphism (metAFLP) [42–44] could be appropriate for their identification.

The metAFLP is dedicated to studying DNA methylation and sequence variation [43, 45]. In contrast, the MSAP identifies DNA methylation variation exclusively. Both techniques identify changes in the DNA methylation pattern, but distinct DNA sequences are being analyzed. The advantages of the metAFLP approach [44], namely quantification of sequence variation (SV), DNA demethylation (DMV) and de novo methylation (DNMV) including symmetric CG, CHG, and asymmetric CHH contexts (where H = A, T, or C) and the fact the approach was successfully used in the case of in vitro tissue culture studies of many species [39, 46–48], predisposes that method to research. The metAFLP is a choice for studies involving different aspects of tissue cultures. One such issue is the optimisation of plant regeneration via somatic embryogenesis [49, 50], which could be performed if an adequate statistical approach is used to simultaneously cope with many factors and minimize experimental efforts [51]. The Taguchi approach [52] of experimental design optimisation could be considered for such purposes. The method has not been widely exploited in the biological studies [53] except for identifying factors affecting germination in barley [54], *Miscanthus sinensis* [55] and in the optimisation of androgenesis in barley [44, 56].

The study aimed to optimise Cu^2+^ and Ag^+^ concentration in tissue culture medium in parallel to the longevity of plant regeneration step at the induction medium using the Taguchi approach involving the metAFLP quantitative characteristics in the case of barley regenerants derived via somatic embryogenesis.

## Results

### Source of explant tissue-donor plants

All donors that were the progeny of double haploid plants derived via anther cultures were morphologically uniform and in the type of source plant materials. The molecular analysis utilized 13 primer pairs (Additional file 1: Table S1), amplifying 335 *Acc65*I/*Mse*I and 326 *Kpn*I/*Mse*I markers. After virtual ‘extraction’ of markers related to DNA methylation pattern change, forty-eight *Acc65*I/*Mse*I-*Kpn*I/*Mse*I markers were evaluated.

Cluster analysis based on *Kpn*I/*Mse*I markers employing Jaccard’s coefficients of similarity showed uniformity of donor plants ranging from 98.6 to 100%, with the majority being identical (Additional file 2: Fig. S1).

However, DNA methylation related markers *Acc65*I/*Mse*I-*Kpn*I/*Mse*I (Additional file 3: Fig. S2) showed that at this level, donor plants were similar in the range from 76 to 100%.

### The metAFLP marker characteristics for donor and regenerants in M1–M9 trials

All regenerants were fertile, and in the shape of donor plants in terms of plant height, leaf shape, color, anther color, plant vigor, spike number, and method of tillering. The metAFLP analyses of donor and regenerants with eight primer pairs (Additional file 1: Table S1) resulted in 192 markers evaluated for all trials (M1–M9) and shared between *Kpn*I/*Mse*I and *Acc65*I/*Mse*I platforms. There were 19 and 14 polymorphic markers amplified in the *Acc65*I/*Mse*I and *Kpn*I/*Mse*I platforms. The polymorphic loci value (*P* *%)* ranged from 0% (M9) to 3.13% (M7) for *Kpn*I/*Mse*I and from 1.04 (M9) to 4.69% (M7) for *Acc65*I/*Mse*I-*Kpn*I/*Mse*I digests. The mean value of *P* *%* was lower for *Kpn*I/*Mse*I (1.16) platform than for the *Acc65*I/*Mse*I-*Kpn*I/*Mse*I (2.78). Similarly, a mean value of Shannon’s information index (*I*) was lower in the case of *Kpn*I/*Mse*I (0.005) than in *Acc65*I/*Mse*I-*Kpn*I/*Mse*I (0.016) derived markers. Also, expected heterozygosity (*He)* and unbiased expected heterozygosity (*uHe*) values were lower for the sequence data (0.003 and 0.004) than for methylation (0.011 and 0.012) (Additional file 1: Table S2).

### Analysis of sequence variation and DNA methylation alternations for donor and regenerants in M1–M9 trials

Regenerants derived via somatic embryogenesis were either identical or differed by 2% as indicated by cluster analysis performed on *Kpn*I/*Mse*I markers. Most of the regenerants derived in the M1, M3, M4, M5, M8, and M9 trials were identical in sequence variation. The highest level of sequence differences (4%) was demonstrated for the donor plant (JDHK) used as a source of tissues for the in vitro plant regeneration and a single M7 regenerant (Additional file 4: Fig. S3).

Agglomeration of regenerants based on the *Acc65*I/*Mse*I-*Kpn*I/*Mse*I markers showed that DNA methylation changes demonstrated that the analyzed plant similarity ranged from ca. 77 to 97%. However, based on bootstrap values, the differences were insignificant except for a single regenerant and the donor plant. The latter differed from regenerants in more than 40%. In contrast to the *Kpn*I/*Mse*I based grouping, the regenerants derived in the M1–M9 trials failed to form close subclusters except for the M8 and M9 ones (Additional file 5: Fig. S4).

Analysis of molecular variance (AMOVA) performed on the metAFLPs amplified on DNAs extracted from regenerants obtained via somatic embryogenesis in the optimisation experiment showed that explained variance among trials (M1–M9) equaled to 0.576 and 0.529 in the case of *Kpn*I/*Mse*I and *Acc65*I/*Mse*I-*Kpn*I/*Mse*I markers, respectively.

### Quantifying the metAFLP-derived variation and ANOVA for M1–M9 trials

Quantitative the metAFLP characteristics, namely TCIV, SV, DMV and DNMV (Table 1) evaluated for the regenerants derived via the M1–M9 trials indicated that in general, SV was higher than DMV and DMV was higher than DNMV. The values of all metAFLP characteristics increased in the M8 and M9 regenerants that grew on the in vitro medium with the highest concentration of copper ions (Table 1). The highest concentration of silver ions and short time of in vitro cultures in the M8 acted the same way as lack of the ions and long-propagated cultures. In general, the regenerants derived under control conditions (M1) exhibited the lowest values of variation except for DNMV.

**Table 1:** MetAFLP quantitative characteristics of the regenerants derived via somatic embryogenesis using tissue culture conditions of the M1–M9 trials and ANOVA results, including Tukey’s HSD tests

		MetAFLP characteristics				Tissue culture conditions		
		TCIV	SV	DMV	DNMV	CuSO_4_ (µM)	AgNO_3_ (µM)	Length of induction step (days)
ANOVA	*F*	32.676	14.612	12.177	7.274			
	*p*	0.0001	0.0001	0.0001	0.0001			
Trials	M1	7.75^c^	2.01^bc^	1.92^c^	1.63^bc^	0.1	0	21
	M2	8.23^c^	2.78^bcd^	1.92^c^	1.34^c^	0.1	10	28
	M3	7.94^c^	2.21^d^	1.92^c^	1.53^c^	0.1	60	35
	M4	7.85^c^	2.11^d^	2.40^b^	1.34^c^	5	60	28
	M5	7.66^c^	2.11^d^	2.40^b^	1.34^c^	5	0	35
	M6	8.13^c^	2.59 ^cd^	2.40^b^	1.63^bc^	5	10	21
	M7	9.67^b^	3.83^a^	2.40^b^	1.53^c^	10	10	35
	M8	10.67^ab^	3.47^ab^	2.41^a^	2.22^ab^	10	60	21
	M9	10.77^a^	3.37^abc^	2.41^a^	2.31^a^	10	0	28

The TCIV, SV, DMV, and DNMV reflect tissue culture-induced variation, sequence variation, demethylation, and *de novo* methylation, respectively. The a, b, and c indicate Tukey’s HSD grouping

Analysis of variance indicated that differences among mean values of all variation types were significant (Table 1). Tukey’s HSD grouping revealed an intricate pattern of grouping regenerants dependent on the metAFLP quantitative characteristic. The TCIV demonstrated that the M1–M6 trials formed the first, M7–M8 the second, and M8–M9 the third group of regenerants. Sequence variation showed that the regenerants of the trials could be grouped into four partly overlapping groups. Also, three somewhat coinciding groups were distinguished with DNMV. Only DMV could evaluate three separate groups of trials with the M8 and M9 forming the first, M4–M7 the second, and M1–M3 the last one (Table 1).

### Taguchi based optimisation

Based on the metAFLPs amplified on DNAs of regenerants extracted from leaves of the M2–M9, using TCIV characteristic subjected to the Taguchi approach, minimum differences between the donor plant and its regenerants should be expected under 10 µM CuSO_4_ and 60 µM AgNO_3_ (the M12 trial) concentrations of the induction medium. When the induction medium was 2.95 µM CuSO_4_, and 15 µM AgNO_3_ (M13), the highest values of differences were predicted. The longevity of in vitro tissue culture evaluated by the Taguchi method was 21 and 28 days for the M12 and M13 optimised trials, respectively.

### Regenerants derived under optimised conditions in M10–M13 trails

The M12 and M13 trials resulting from Taguchi-based optimisation of tissue culture conditions of plant regeneration via somatic embryogenesis resulted in regenerants that were identical with the donor plant and control regenerants (M10). Regenerants from trials M12 and M13 exhibited any morphological differences in assessed traits (plant height and vigor, leaf shape and color, anther color, plant vigor, spike number, and method of tillering).

### Analysis of sequence variation and DNA methylation alternations for donor and regenerants in M10–M13 trails

The metAFLP analyses based on DNAs of donor plant and regenerants were performed on eight primers pair (Additional file 1: Table S2) resulted in 195 markers shared between *Kpn*I/*Mse*I and *Acc65*I/*Mse*I platforms among the M10, M12, and M13 trials. The *Acc65*I/*Mse*I amplified 49 and *Kpn*I/*Mse*I 26 polymorphic DNA fragments.

Agglomeration analysis of regenerants derived via the M10, M12, and M13 trials and based on the *Kpn*I/*Mse*I markers (Additional file 6: Fig. S5) demonstrated that the donor plant was separated from all regenerants that formed two major clusters. The first one consisted of five regenerants from the M10 trial, whereas the second subcluster encompassed all other regenerants. Although further subclustering of the data was insignificant, the regenerants of the M13 trial (optimised towards maximum differences relative to the donor plant) exhibited the highest level of sequence variation with a similarity of about 97%. The M12 (towards minimum differences relative to the donor plant) regenerants were nearly identical at the sequence variation level (~ 99% of similarity) and were similar to the remaining M10 regenerants.

Grouping with *Acc65*I/*Mse*I-*Kpn*I/*Mse*I markers (Additional file 7: Fig. S6) revealed higher than *Kpn*I/*Mse*I markers level of differences among regenerants with the donor plant being apart of them. No significant clustering was evaluated. However, the M12 regenerants, in contrast to the M13, were much more similar to each other. Furthermore, the M10 plants seemed to be the most uniform (except in two cases) and close to the M12 regenerants.

Analysis of molecular variance (AMOVA) performed on the *Kpn*I/*Mse*I (K) markers revealed that the explained variance among regenerants of the trials equaled to 49.8% (Φ_*PT*_ value = 0.498, *p *= 0.001) whereas in the case of *Acc65*I/*Mse*I-*Kpn*I/*Mse*I (M) markers the respective variance equaled to 34.6% (Φ_*PT*_ value = 0.346, *p *= 0.001).

### Quantifying the metAFLP-derived variation and ANOVA for M10–M13 trials

Based on the TCIV, the Grubbs test showed that regenerant number two (M10) and seven (M13) were outliers and were removed from the analysis (data not shown).

Quantification of the metAFLP characteristics (TCIV, SV, DMV, DNMV) obtained for regenerants derived under optimised conditions showed that the SV was slightly higher than the DMV (Table 2). In contrast, methylation was more than three times lower than SV. The mean value for DNMV and DMV demonstrates that demethylation prevailed over de novo methylation in all trials. The highest values of the TCIV were in the M13 trial (under the highest concentration of CuSO_4_-maximum differences conditions). The lowest values of differences in TCIV were in the M12 (minimum differences between donor and regenerants). TCIV characteristics for the M10 (control) trial were in between those for the M12 and M13. The DNMV was the highest for the M13 and the lowest for the M12. Although DMV for M13 was higher than for the M12, under M10 control conditions, DMV was the highest. Interestingly, SV was the highest for the M10 and the lowest for M13.

**Table 2:** The arrangement of ANOVA statistics and Tukey’s HSD tests for trials in the verification process

		MetAFLP characteristics				Tissue culture conditions		
		TCIV	SV	DMV	DNMV	CuSO_4_ (µM)	AgNO_3_ (µM)	Length of induction step (days)
ANOVA	F	9.117	0.192	16.997	7.009			
	*p*	0.001	0.827	0.0001	0.004			
Trial	M10	12.76^a^	4.34^a^	4.41^a^	1.26^ab^	0.1	0	21
	M12	11.01^b^	4.22^a^	3.65^c^	0.72^b^	10	60	21
	M13	13.66^a^	4.10^a^	3.98^b^	2.26^a^	2.95	15	28

M10 states for control, whereas M12-M13 for the verification trials. TCIV, SV, DMV, and DNMV, reflect tissue culture-induced variation, sequence variation, demethylation, and *de novo* methylation. The a, b, and c indicate Tukey’s HSD grouping. The presented data does not include outliers that were excluded based on the Grubs test

Based on ANOVA, all but the SV metAFLP characteristics showed significant differences between tested trials M10, M12, and M13 (Table 2). According to Tukey’s HSD test, the M10 and M13 formed a single group, whereas the M12 remained an individual based on the TCIV. Grouping arranged for DMV divided trials into three distinct groups. The DNMV based grouping creates two groups share for M10, M13, and M10, M12 trials. ANOVA underlines the most variabilities for comparing M12 and M13 trials.

### Methylation contexts in M10–M13 trials

The comparison made for regenerants from trials in a verification experiment concerning demethylation and de novo methylation indicated that DMV was most often observed in the context of CHG. DNMV in the context of CHG was four times less frequent. De novo methylation and demethylation in the CG methylation context were lower than in the CHG context. The changes in DNA methylation in the asymmetric CHH context concerned only de novo methylation. In all tested contexts, the highest values of either DMV or DNMV were in the M13 trial (Table 3).

**Table 3:** The ANOVA and Tukey’s HSD test for different statistical models by methylation contexts and within DNMV and DMV

Methylation context		CG		CHG		CHH	
MetAFLP characteristics		DMV	DNMV	DMV	DNMV	DMV^*^	DNMV
ANOVA	F	3.337	33.951	54.124	1.992	–	132259.418
	*p*	0.052	0.0001	0.0001	0.158	–	0.0001
Trials	M10	0.26^ab^	0.00^b^	4.15^a^	0.66^a^	–	0.61^a^
	M12	0.10^b^	0.10^b^	3.56^b^	0.62^a^	–	0.00^b^
	M13	0.41^a^	0.87^a^	3.59^b^	1.39^a^	–	0.00^b^

The M10, M12, and M13 state for trials, whereas upper case letters reflect Tukey’s HSD grouping. *ANOVA not available for 0 value

ANOVA was significant in the case of CG and CHH contexts for de novo methylation. In DNA demethylation, only the CHG context was significant, while the CG one was close to significance. Tukey’s HSD test differentiated between the M10, M12, and M13 trials so that either the M10 or M13 formed separate groups or were one of the trials located in the same group with M12 in the case of significant ANOVA results (Table 3).

## Discussion

Efforts to optimise plant regeneration via in vitro cultures began almost with the initiation of plant tissue culture techniques [57]. Initially, these works led to the compilation of appropriate ingredients forming the growing media. In this way, even commonly used media such as MS [58] or Gamborg B5 medium [59] have been created. Although many species responded positively to MS medium, many of them required more sophisticated compounds. Thus, from the beginning of plant tissue cultures to nowadays, the regeneration conditions are being improved. With the development of knowledge on tissue cultures, it has been proven that the conditions used in vitro may lead to changes in regenerated plants. Apart from morphological [60] also genetic [61] and epigenetic [39, 42, 62] alterations between regenerants and donor plants are observed and quantified by the metAFLP approach [42]. The metAFLP approach showed that it is sufficiently informative for analysis of even closely related species as well as tissue culture-derived regenerants [63, 64]. Shannon’s Indices were low in the given study, ranging from 0.000 to 0.18 and 0.007 to 0.023 in *Kpn*I/*Mse*I and *Acc65*I*/Mse*I-*Kpn*I/*Mse*I data sets, respectively. Such a result was expected as all regenerants originated from a single, double haploid donor plant. A low level of variation was confirmed by percentages of polymorphic loci (*P* *%*), hetero- (*He*), and expected heterozygosity (*uHe*) with increased for the *Acc65*I/*Mse*I-*Kpn*I/*Mse*I data. Presented results are congruent with those published by the others where metAFLP model was successfully applied in studies devoted to cereals [42, 44, 64–66] and other plants: *Armeria maritima s.l.* [67], *Arabidopsis thaliana* [48], *Gentiana pannonica* Scop [46], and *Gentiana kurroo* [68].

To fit the metAFLP analysis model, donor plants need to be uniform at genetic and epigenetic levels. The donor plants were the generative progeny of regenerants obtained via androgenesis as generative cycles may stabilize genetic and epigenetic alterations induced during in vitro plant regeneration [39, 44]. Cluster analysis demonstrated the genetic and epigenetic uniformity of the donors reached up to 100%. However, DNA methylation-related variation was higher than genetic one what may reflect demethylation and de novo methylation processes required for plant regeneration via tissue cultures during the cell reprogramming stage [69, 70]. The cell reprogramming process is error-prone and not necessarily leads to a donor plant’s identity and its regenerants. Thus, a randomly chosen donor double haploid plant after a single generative cycle was used as a source of explants for the optimisation step and another plant that originated from the same progeny for the verification experiments. The approach should minimize the donor’s effect as it may impact regenerated plants [71, 72].

It was shown that under optimisation experimental conditions, DNA methylation alternations were usually higher than sequence changes in the case of barley genotypes. On the other hand, in triticale, genetic changes prevailed [73]. Thus, the preference may depend on species. Also, medium ingredients, such as copper and silver ions, may influence (epi)genetic variation. In barley anther culture, the two ions may act as mediators of sequence variation that involves different methylation contexts [74]. They may also affect green plant regeneration [75] and somewhat differently act in somatic embryogenesis [76]. Thus, playing with the concentration of copper and silver ions in the induction medium may influence the variation level. That is what was precisely observed in an optimisation experiment. Depending on the trials’ experimental conditions, the fluctuations in SV, DNMV, and DMV were demonstrated underlined by cluster analysis and supported by AMOVA, suggesting that more than 50% of the explained variance was due to trial differences. In general, with the increased concentration of copper ions, the upsurge of all metAFLP characteristics was observed. The role of silver ions was related to the time of tissue cultures. The ions higher concentration was significant only when a short time of cultures was used (M8); however, when the time increased, the lack of silver ions resulted in comparable levels of TCIV variation detected in the M8–M9 trials. The observed differences in TCIV in optimisation experiments may be related to some problems with the electron transport chain (complex I and III) that involves Cu/Zn dismutase. In the presence of copper ions, *superoxide dismutase* functioning might be disturbed, influencing the Yang cycle [77–79]. The notion is confirmed by the fact that TCIV is composed of SV, DNMV, and DMV. Furthermore, DNMV and DMV were predictors leading to SV using copper ions as mediators in andro- [74] and embryogenesis [76]. Besides, copper ions participate in oxidative stress and may modify methylated cytosines leading to SV [80]. In this context optimisation of tissue culture conditions towards maximum and minimum variation is of the highest priority.

Using the Taguchi method and TCIV evaluated for the M2–M9 trials, the in vitro conditions leading towards either minimum or maximum differences between the donor plant and its regenerants were proposed. The differences were mainly due to the concentration of CuSO_4_. The differences between the trials are also highlighted in the cluster analysis. Explained molecular variance related to sequence changes for the regenerants of the M10–M13 trials was close to 50% (as in the optimisation experiment). In contrast, the variation due to methylation pattern equaled 35%-it suggested that sequence changes in the optimisation and verification experiments were nearly at the same level. When the respective regenerants were tested using the metAFLP approach, it was shown that the trials differed among each other and that in contexts of TCIV, the optimisation resulted in expected results. The M13 regenerants exhibited higher than the M12 level of DMV and DNMV. Furthermore, the M12 and M13 trials were grouped in opposite groups based on Tukey’s HSD test. While in all cases DNMV methylation related changes exceeded those for DMV, the Taguchi method suggested in vitro culture conditions under which the level of TCIV was optimised in the expected direction compared to the control conditions. It was also demonstrated that the within trial variation was higher for the regenerants derived under the M13 conditions compared to the M12, whereas the M12 were closer to the M10 or M10 was in between M12 and M13. The presented data is coherent with earlier results devoted to optimising barley anther culture where differences between optimised towards maximum and minimum differences trials were evaluated [44].

A remarkable aspect of the study is that the metAFLP approach can identify DNA methylation changes affecting all sequence methylation contexts [44]. Differences in DNA methylation context were illustrated using regenerants derived on optimised tissue culture conditions. Depending on the sequence context, either DNMV or DMV were responsible for the grouping of optimised trials. ANOVA showed that trials (M10–M13) differed considering the DNA de novo methylation in CG and CHH methylation contexts. A similar comparison between trials (M10–M13) carried for DNA demethylation showed significant differences only for CHG methylation context. Thus, problems with S-adenosyl-l-methionine compound, which is responsible for DNMV, were crucial in differentiating trials. Remarkably, DNMV of the CG contexts was significant with the highest value for the M13 compared to the other trials, whereas DNMV of the CHH sequence was the highest for the M10 control trial. The CG context is hardly stable in terms of methylation [81], and the methylation pattern is reestablished via replication mechanisms [82]. On the other hand, the CHH context is regulated epigenetically via, e.g., RNAi mechanisms [83]. Assuming that the in vitro tissue cultures lead to many DNA methylation changes due to the in vitro-induced stresses, it is entirely reasonable to assume that the CG and CHH contexts should be less affected by changes than the CHG as the methylation of the latter is regulated by replication and epigenetic mechanisms [84], as it was demonstrated in presented data. Possibly higher DMV than DNMV of the CHG contexts reflects the demethylation stage followed by only partial DNA methylation pattern recovery [69, 70]. On the other hand, slightly increased de novo methylation of the CHH sequences may reflect either experimental issues to detect subtle changes or real phenomena related to methylation contexts. Assuming that many metAFLP markers were analyzed, experimental artifacts could be excluded. What was observed reflects not fully recognized mechanisms affecting in vitro tissue culture plant regeneration at the DNA methylation level.

The presented data shows that DNA demethylation (both in optimisation and verification experiments) dominates over de novo methylation independently of the DNA methylation sequence context analyzed (except CHH where DMV was not detected). Similar data were obtained in previous studies on barley [42] and triticale regenerants [39] from anther cultures. Interestingly, the CHG context is the most affected one by DMV. Possibly, the cell reprogramming step [85] involving active [86, 87] and passive [88, 89] DNA demethylation followed by different mechanisms of de novo methylation (depending on sequence contexts) [90, 91] and variations in the preferences towards methylation of specific cytosines compared to methylation density of some genomic regions [92] and correctness of pattern reestablishment due to (epi)genetic mechanisms [93] may be the most probable explanation both for the increased values of DMV and preferential demethylation of the CHG sequences.

## Conclusions

Presented research showed that by manipulating the concentration of the in vitro tissue culture medium ingredients such as copper and silver ions and the time of tissue cultures, it is possible to regenerate plants that are either close to the source of explant or vice versa at the level of the tissue culture-induced variation. Furthermore, using the metAFLP approach combined with the Taguchi method, the in vitro tissue culture plant regeneration could be optimised in the expected direction. Moreover, the metAFLP approach may quantify methylation changes affecting symmetric and asymmetric sites. Most of the methylation changes evaluated in the verification experiment were related to the DMV of the CHG contexts; however, the reason for such a preference is not apparent.

## Methods

### Donor plant derivation

Spring barley cultivar NAD2 accessible from Poznan Plant Breeders LTD-Nagradowice, (Poland) served as source material to prepare donor plants via in vitro anther culture [44]. The diploidisation of haploid regenerants carried spontaneously. Doubled haploid regenerants were used to obtain generative offspring, which served as donor plants for regenerant production via somatic embryogenesis from immature zygotic embryo culture for the optimisation and verification process based on the Taguchi method. Thirty-six donor plants were derived for optimisation and verification experiments.

### Optimisation

#### Plant material and sterilization

Twenty-four donor plants were cultivated in controlled conditions in the growth chamber. Collection of spikes with unripe caryopses were done after 12-16 days of self-pollination. After dissection, the caryopses were surface-sterilized in 70% ethanol for 1 min and transferred to 10% sodium hypochlorite solution (NaOCl) for 20 min with stirring. Next, caryopses were thoroughly rinsed four times with sterile water. From disinfected caryopses, immature zygotic embryos were extracted and plated on solid N6L medium containing macro- and micronutrients [94] with 2 mg l^−1^ 2,4-dichlorophenoxyacetic acid (2,4-D), 0,5 mg l^−1^ 6-naphthalene acetic acid (NAA), and 0.5 mg l^−1^ kinetin.

#### Callus induction

Induction media based on N6L were prepared in nine combinations (M1–M9) according to the Taguchi approach, supplemented with CuSO_4_ and AgNO_3_ at varying concentrations (Table 1). The time of induction step was counted from plating immature embryos on induction media to transferring calli, first somatic embryos and embryo-like structures on regeneration media. Immature zygotic embryos were incubated for callus and somatic embryos induction at 26 °C under photoperiod 16 h light/8 h dark. Primary explant derived calluses, somatic embryos, and embryo-like structures were observed several days later.

#### Somatic embryogenesis conditions

The regeneration step was conducted on solid medium K4NB with 0.225 mg l^−1^ 6-benzylaminopurine (BAP) [95]. The calli and embryo-like structures were incubated at 26 °C under a 16 h light/8 h dark photoperiod. Regenerated plantlets were put into flasks with induction N6I rooting medium [94] supplemented with 2 mg l^−1^ indole-3-acetic acid (IAA). Next, regenerants were potted to the soil-sand mixture (3:1) and were grown to maturity under controlled conditions in a greenhouse on the abovementioned day/night regime. Forty-five regenerants derived via somatic embryogenesis prepared in nine trials (M1–M9) derived from one donor plant were ready for optimisation experiment.

### Verification

After establishing optimised conditions, the selected levels of used factors (CuSO_4_, AgNO_3_, length of induction step) were verified by new regeneration plants from immature zygotic embryo cultures. The somatic embryogenesis was established on explants from 12 donor plants. The essential components of induction media were prepared for the optimisation process with changed levels of tested factors (Table 2). A total of three trials were performed: M10-control, M12-verification towards the lowest percentage of differences based on Tissue Culture Induced Variation (TCIV) between donor and regenerants, and M13 verification towards the highest percentage of differences between donor and regenerants (Table 2). The regeneration media in the verification step were the same as for the optimisation step. All regenerants obtained in three trials (M10, M12, M13) were derived from a single donor plant in the verification process.

### DNA preparation and metAFLP analysis

The genomic DNA was isolated from 100 mg of fresh leaves of donor plants and regenerants obtained via somatic embryogenesis from optimisation and verification processes using the DNasy MiniPrep kit (Qiagen, Hilden, Germany). The metAFLP was used as described earlier [42] with slight modifications [43]. The genomic DNA was subjected to cutting with two pairs of enzymes *Acc65*I/*Mse*I and *Kpn*I/*Mse*I. The subsequent metAFLP steps (adaptor ligation, pre-selective, and selective amplification steps) were performed using dedicated adaptors and primers (Additional file 1: Table S3). The completion of all stages of the metAFLP technique resulted in obtaining a band profile of DNA fragments. The bands’ profile obtained with cutting DNA with the *Acc65*I/*Mse*I and *Kpn*I/*Mse*I platforms, expressed as 0–1 matrices (absence-presence), were contrasted. This combination of data, where the platform *Acc65*I/*Mse*I, which concerns sequence changes and DNA methylation with data from *Kpn*I/*Mse*I platform, results in information about the DNA sequence, allows for the extraction of only methylation data. Simply, markers that were present in the first and missed in the second (or vice versa) metAFLP platform were related to DNA methylation [63]. Thus, the data from the *Acc65*I/*Mse*I-*Kpn*I/*Mse*I were used to evaluate the “methylation markers”, whereas those from the *Kpn*I/*Mse*I one were the source of information concerning sequence variation. Data obtained from both metAFLP platforms were used to assess the quantitative metAFLP characteristics described elsewhere [42, 43]. Among others, the tissue-culture induced variation (TCIV), sequence variation (SV), demethylation (DMV), and de novo methylation (DNMV), were assessed. Based on specially designed selective primers to metAFLP, it was possible to estimate changes related to CG and CHG methylation (symmetric contexts) and CHH (asymmetric context). Thus, events identified using markers amplified by selective primers directed towards symmetric and asymmetric contexts could be evaluated as described earlier [44].

### Statistics

The molecular markers obtained in both metAFLP platforms were subjected to GenAlEx6.501 (Excel add-in software) [96] to estimate a percentage of polymorphic loci (*%P*), Shannon’s information index (*I*), expected heterozygosity (*He*), and unbiased expected heterozygosity (*uHe*).

UPGMA based on the Jaccard index was conducted in PAST software [97]. The significance of grouping was evaluated using 999 bootstrap replicates.

The analysis of molecular variance (AMOVA) conducted using the sequence and methylation metAFLP markers evaluated based on *Kpn*I/*Mse*I and *Acc65*I/*Mse*I-*Kpn*I/*Mse*I AFLP platforms in GenAlEx6.501 software allowed assessing *Φ*_*PT*_ index values.

Qualitative and quantitative metAFLP characteristics were evaluated as described earlier [42–44].

The outliers were identified using metAFLP TCIV in XLSTAT 2018.1.49, 205 software (Addinsoft, Paris, France). The Grubbs’ test at a significance level of 5% was performed.

The Taguchi approach towards the maximum and the minimum number of differences between donor plants and regenerants was conducted using TCIV characteristics evaluated based on the M2-M9 trials in QI Macros230T (KnowWare International, Inc, Denver, USA).

ANOVA and Tukey’s HSD groupings at *p *< 0.05 were used to evaluate differences among trials using the TCIV, SV, DMV, DNMV, and symmetric and asymmetric metAFLP characteristics in XLSTAT 2018.1.49205 Excel add-in software.

## Supplementary information

**Additional file 1: Table S1.** Primers used in metAFLP for donors and regenerants in optimisation and verification experiments. **Table S2.** The indices characterizing *KpnI*/*MseI* and *Acc65*I/*MseI*-*KpnI*/*MseI* metAFLP marker systems evaluated for regenerants derived *via* somatic embryogenesis. **Table S3.** Oligonucleotides applied for metAFLP in barley studies.

**Additional file 2: Figure S1.** Agglomeration analysis based on the sequence markers *KpnI*/*MseI* for donor plants: JDH1-JDH24.The bootstrap values are indicated at the nodes.

**Additional file 3: Figure S2.** Agglomeration analysis based on virtually derived markers (*Acc65*I/*MseI*-*KpnI*/*MseI*) reflecting DNA methylation difference between JDH1–JDH24 donor plants. The bootstrap values are indicated at the nodes.

**Additional file 4: Figure S3.** Agglomeration analysis (UPGMA, Jaccard) based on *Kpn*I/*Mse*I markers for regenerants derived *via* somatic embryogenesis *via* M1–M9 trials and their JDHK donor plant. The bootstrap values are marked at the nodes.

**Additional file 5: Figure S4.** Agglomeration analysis (UPGMA, Jaccard) based on *Acc65*I/*MseI*-*KpnI*/*MseI* virtual markers related to DNA methylation differences evaluated for regenerants derived *via* somatic embryogenesis in the M1–M9 trials and JDHM-donor plant. The bootstrap values are marked at the nodes.

**Additional file 6: Figure S5.** Agglomeration analysis (UPGMA, Jaccard) conducted on the metAFLPs related to the DNA mutations *KpnI*/*MseI* for the M10-M13 trials. The M10 reflects control conditions, whereas the M12 and M13 regenerants derived according to the optimised conditions directed towards the minimum and maximum differences between donor and regenerants. Bootstrap value is indicated on the nodes.

**Additional file 7: Figure S6.** Agglomeration analysis (UPGMA, Jaccard) conducted on the metAFLPs related to the DNA methylation changes (*Acc65*I/*MseI*-*KpnI*/*MseI*) for the M10-M13 trials. The M10 reflects control conditions, whereas the M12 and M13 regenerants derived according to the optimised conditions directed towards the minimum and maximum differences between donor and regenerants. Bootstrap value is indicated on the nodes.

## Data Availability

The datasets during and/or analysed during the current study available from the corresponding author on reasonable request.

## Abbreviations

2,4-D: 2,4-dichloropchenoxyacetic acid
BAP: 6-benzylaminopurine
MS: Murashige and Skoog basal salt mixture
MSAP: Methylation sensitive amplified polymorphism
metAFLP: Methylation sensitive amplified fragment length polymorphism
NAA: 6-naphthalene acetic acid
