# Supplementary material for: Barley somatic embryogenesis-an attempt to modify variation induced in tissue culture
Source: J Biol Res (Thessalon). 2021 Mar 16;28:9. doi: 10.1186/s40709-021-00138-5 (PMC7962293; doi:10.1186/s40709-021-00138-5)
Supplement: Supplementary file 1 — Additional file 1: Table S1. Primers used in metAFLP for donors and regenerants in optimisation and verification experiments. Table S2. The indices characterizing KpnI/MseI and Acc65I/MseI-KpnI/MseI metAFLP marker systems evaluated for regenerants derived via somatic embryogenesis. Table S3. Oligonucleotides applied for metAFLP in barley studies. [file 40709_2021_138_MOESM1_ESM.docx]

**SUPPLEMENTARY MATERIAL**

**Table S1.** Primers used in metAFLP for donors and regenerants in optimisation and verification experiments.

|  | Plant material | |
| --- | --- | --- |
| Primers combinations | Donors | Regenerants |
| CG-GAC/MCGT | 1 | 1 |
| CG-GCA/MCGT | 1 | - |
| CG-GGC/MCAC | 1 | 1 |
| CG-TCG/MCAC | 1 | - |
| CXG-AGA/MCGT | 1 | 1 |
| CXG-AGC/MCAC | 1 | 1 |
| CXG-AGG/MCGT | 1 | 1 |
| CXG-ATG/MCGT | 1 | - |
| CXG-TGC/MCGT | 1 | - |
| CXG-TTG/MCAC | 1 | - |
| CXG-TTG/MCGT | 1 | 1 |
| CXX-ATT/MCAC | 1 | 1 |
| CXX-TAA/MCGT | 1 | 1 |
| Total | 13 | 8 |

**Table S2** The indices characterizing KpnI/MseI and Acc65I/MseI-KpnI/MseI metAFLP marker systems evaluated for regenerants derived *via* somatic embryogenesis.

| metAFLP | *KpnI*/*MseI* | | | | *Acc65*I/*MseI*-*KpnI*/*MseI* | | | |
| --- | --- | --- | --- | --- | --- | --- | --- | --- |
| Trial | *P%* | *I* | *He* | *uHe* | *P%* | *I* | *He* | *uHe* |
| M1 | 0.52 | 0.004 | 0.003 | 0.003 | 3.13 | 0.020 | 0.014 | 0.015 |
| M2 | 1.56 | 0.008 | 0.005 | 0.006 | 3.65 | 0.020 | 0.013 | 0.015 |
| M3 | 1.56 | 0.005 | 0.003 | 0.003 | 3.13 | 0.019 | 0.013 | 0.014 |
| M4 | 1.04 | 0.004 | 0.002 | 0.002 | 2.08 | 0.014 | 0.010 | 0.011 |
| M5 | 1.04 | 0.004 | 0.002 | 0.002 | 2.60 | 0.015 | 0.010 | 0.011 |
| M6 | 1.04 | 0.005 | 0.003 | 0.003 | 2.60 | 0.013 | 0.009 | 0.010 |
| M7 | 3.13 | 0.018 | 0.012 | 0.014 | 4.69 | 0.023 | 0.015 | 0.017 |
| M8 | 0.52 | 0.002 | 0.001 | 0.001 | 2.08 | 0.011 | 0.007 | 0.008 |
| M9 | 0.00 | 0.000 | 0.000 | 0.000 | 1.04 | 0.007 | 0.005 | 0.006 |
| Mean | 1.16 | 0.005 | 0.003 | 0.004 | 2.78 | 0.016 | 0.011 | 0.012 |

P% - a percentage of polymorphic loci, I - Shannon's information index. M1 states for a control experiment, whereas the M2-M9 are the experimental trials

**Table S3.** Oligonucleotides applied for metAFLP in barley studies.

| metAFLP oligomer | Sequence 5’→3’ |
| --- | --- |
| Adaptors |  |
| Ad1 *Acc65*I | CTC GTA GCA TGC GTA CA |
| Ad2 *Acc65*I | GTA CTGTACGCATGCTAC |
| Ad1 *KpnI* | CTC GTA GCA TGC GTA CAG TAC |
| Ad2 *KpnI* | TGTACGCATGCTAC |
| Ad1 *MseI* | TAC TCA GGA CTC ATC |
| Ad2 *MseI* | GAC GAT GAG TCC TGA G |
| Preselective primers | GAT GAG TCC TGA GTA AC |
| Presel Acc56I/*KpnI* | GCA TGC GTA CAG TAC C |
| Presel *MseI* | GAT GAG TCC TGA GTA AC |
| Labeled ^32^P selective oligonucleotides |  |
| CG-GAC | CA TGC GTA CAG TAC CGA C |
| CG-GCA | CA TGC GTA CAG TAC CGC A |
| CG-GGC | CA TGC GTA CAG TAC CGG C |
| CG-TCG | CA TGC GTA CAG TAC CTC G |
| CXG-AGA | CA TGC GTA CAG TAC CAG A |
| CXG-AGC | CA TGC GTA CAG TAC CAG C |
| CXG-AGG | CA TGC GTA CAG TAC CAG G |
| CXG-ATG | CA TGC GTA CAG TAC CAT G |
| CXG-TGC | CA TGC GTA CAG TAC CTG C |
| CG-TTG | CA TGC GTA CAG TAC CTT G |
| CXX-ATT | CA TGC GTA CAG TAC CAT T |
| CXX-TAA | CA TGC GTA CAG TAC CTA A |
| Selective oligonucleotides |  |
| M-CAC | GAT GAG TCC TGA GTA ACA C |
| M-CGT | GAT GAG TCC TGA GTA ACG T |
| M-CTA | GAT GAG TCC TGA GTA ACT A |
