# Supplementary figures and images for: Barley somatic embryogenesis-an attempt to modify variation induced in tissue culture
Source: J Biol Res (Thessalon). 2021 Mar 16;28:9. doi: 10.1186/s40709-021-00138-5 (PMC7962293; doi:10.1186/s40709-021-00138-5)

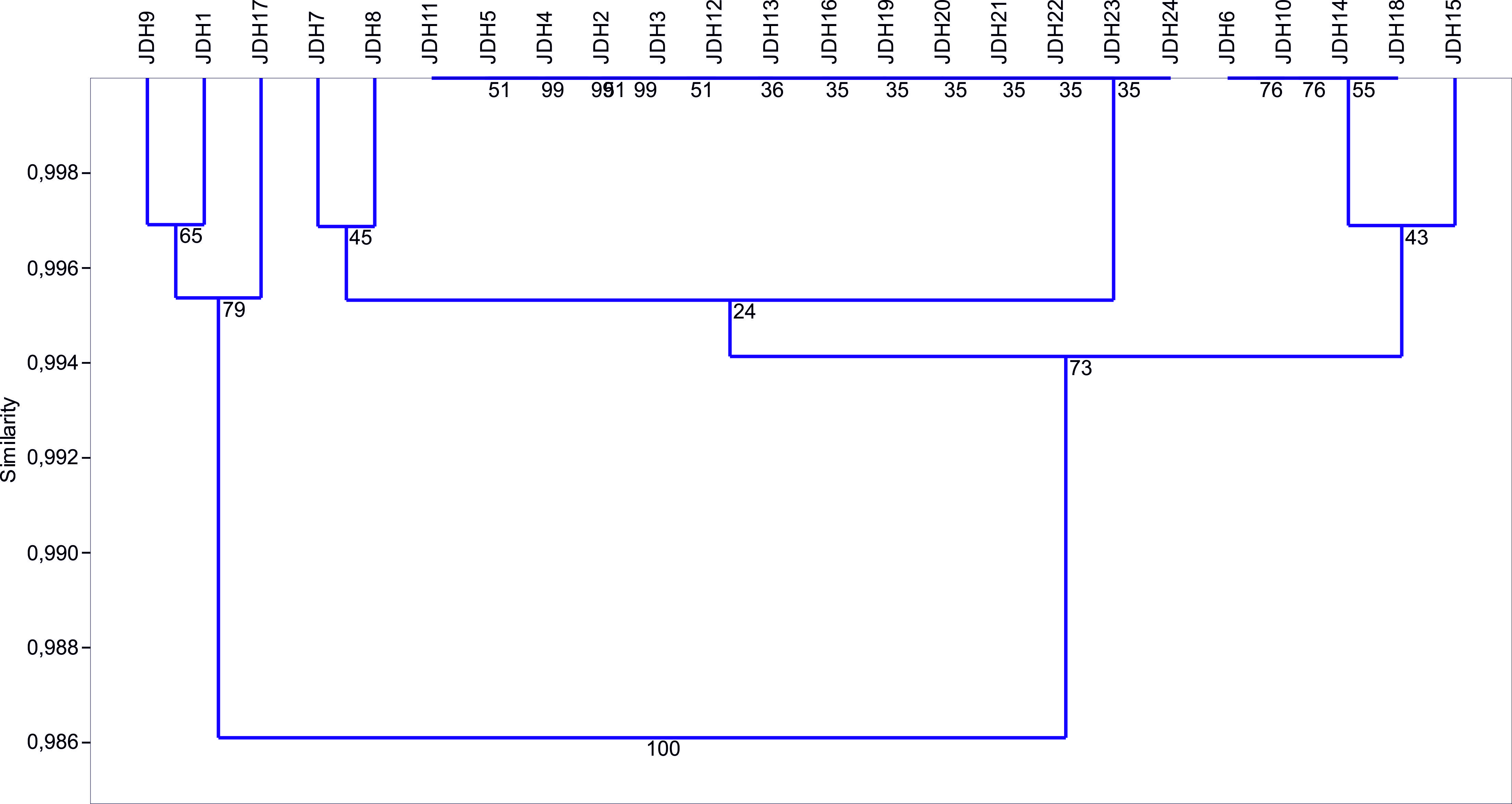

Supplement: Supplementary file 2 — Additional file 2: Figure S1. Agglomeration analysis based on the sequence markers KpnI/MseI for donor plants: JDH1-JDH24.The bootstrap values are indicated at the nodes. [file 40709_2021_138_MOESM2_ESM.jpg]

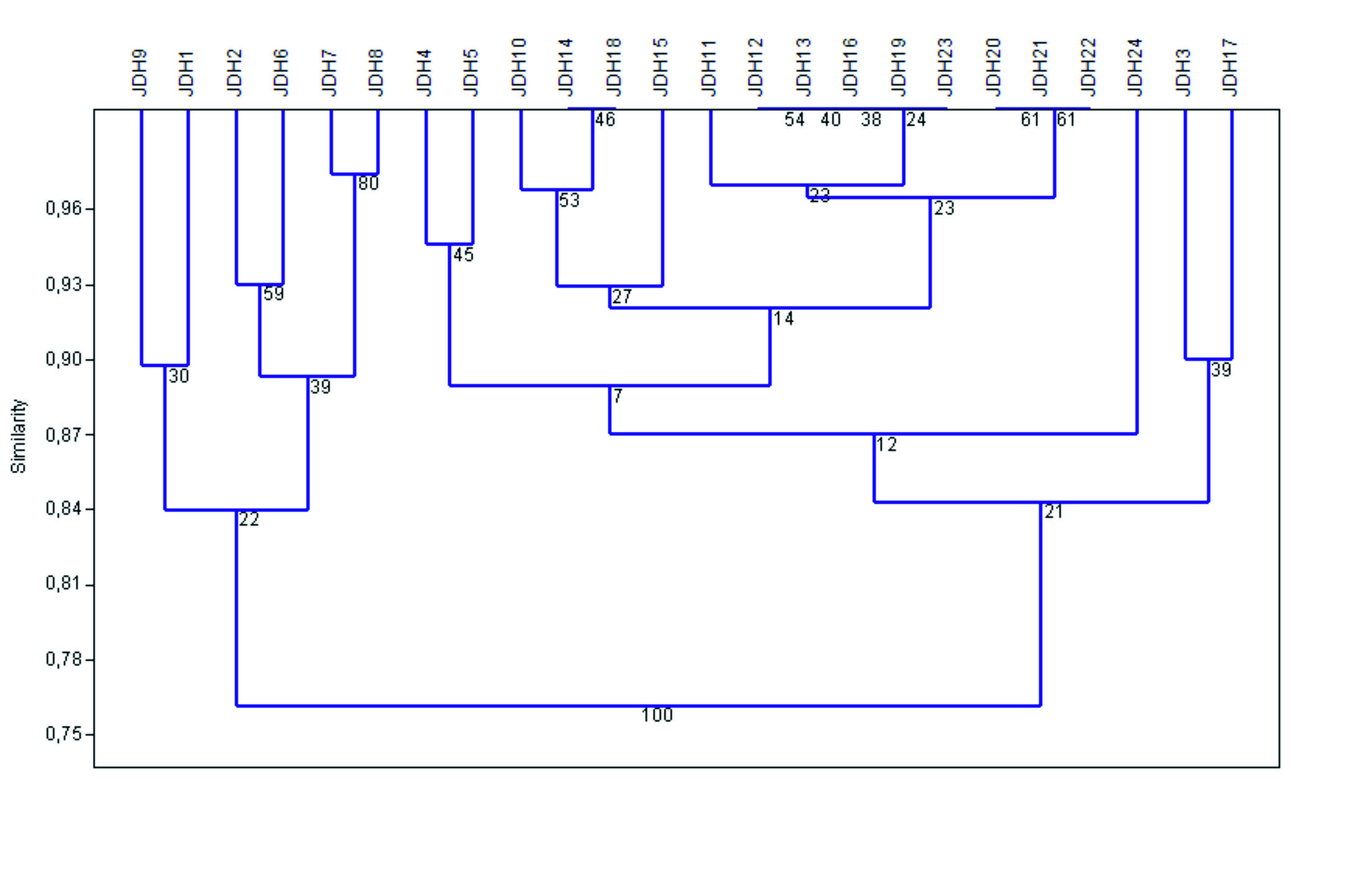

Supplement: Supplementary file 3 — Additional file 3: Figure S2. Agglomeration analysis based on virtually derived markers (Acc65I/MseI-KpnI/MseI) reflecting DNA methylation difference between JDH1–JDH24 donor plants. The bootstrap values are indicated at the nodes. [file 40709_2021_138_MOESM3_ESM.jpg]

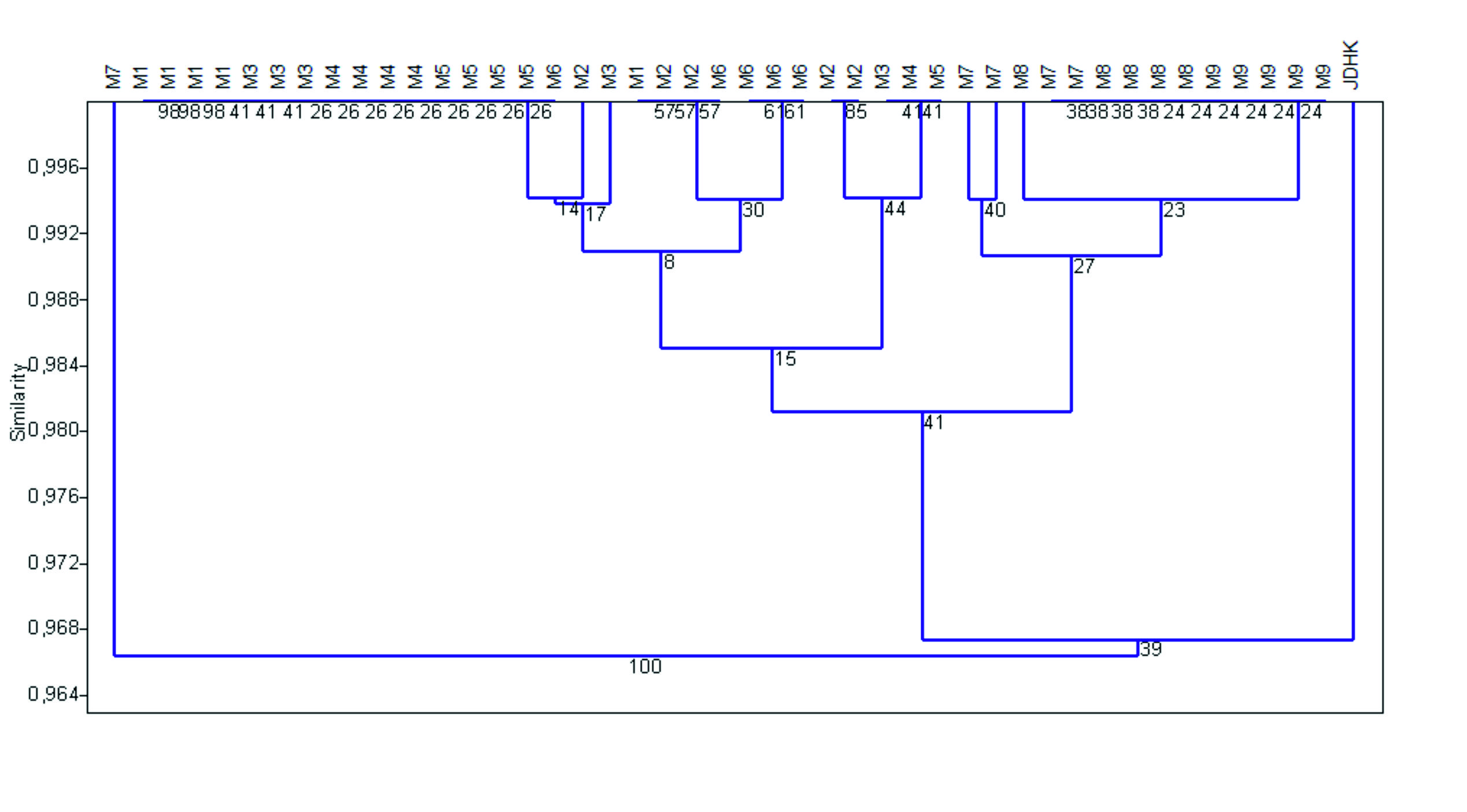

Supplement: Supplementary file 4 — Additional file 4: Figure S3. Agglomeration analysis (UPGMA, Jaccard) based on KpnI/MseI markers for regenerants derived via somatic embryogenesis via M1–M9 trials and their JDHK donor plant. The bootstrap values are marked at the nodes. [file 40709_2021_138_MOESM4_ESM.jpg]

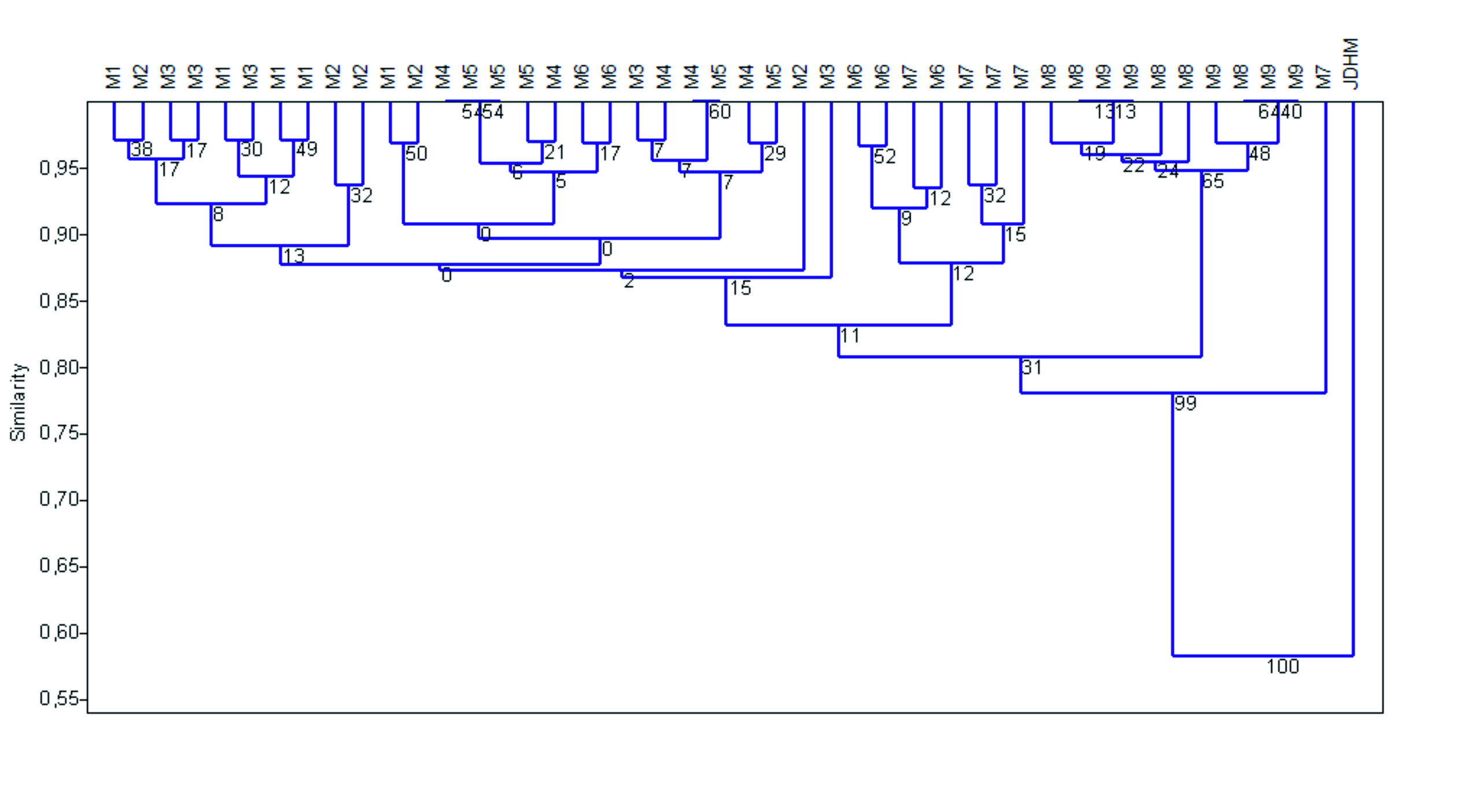

Supplement: Supplementary file 5 — Additional file 5: Figure S4. Agglomeration analysis (UPGMA, Jaccard) based on Acc65I/MseI-KpnI/MseI virtual markers related to DNA methylation differences evaluated for regenerants derived via somatic embryogenesis in the M1–M9 trials and JDHM-donor plant. The bootstrap values are marked at the nodes. [file 40709_2021_138_MOESM5_ESM.jpg]

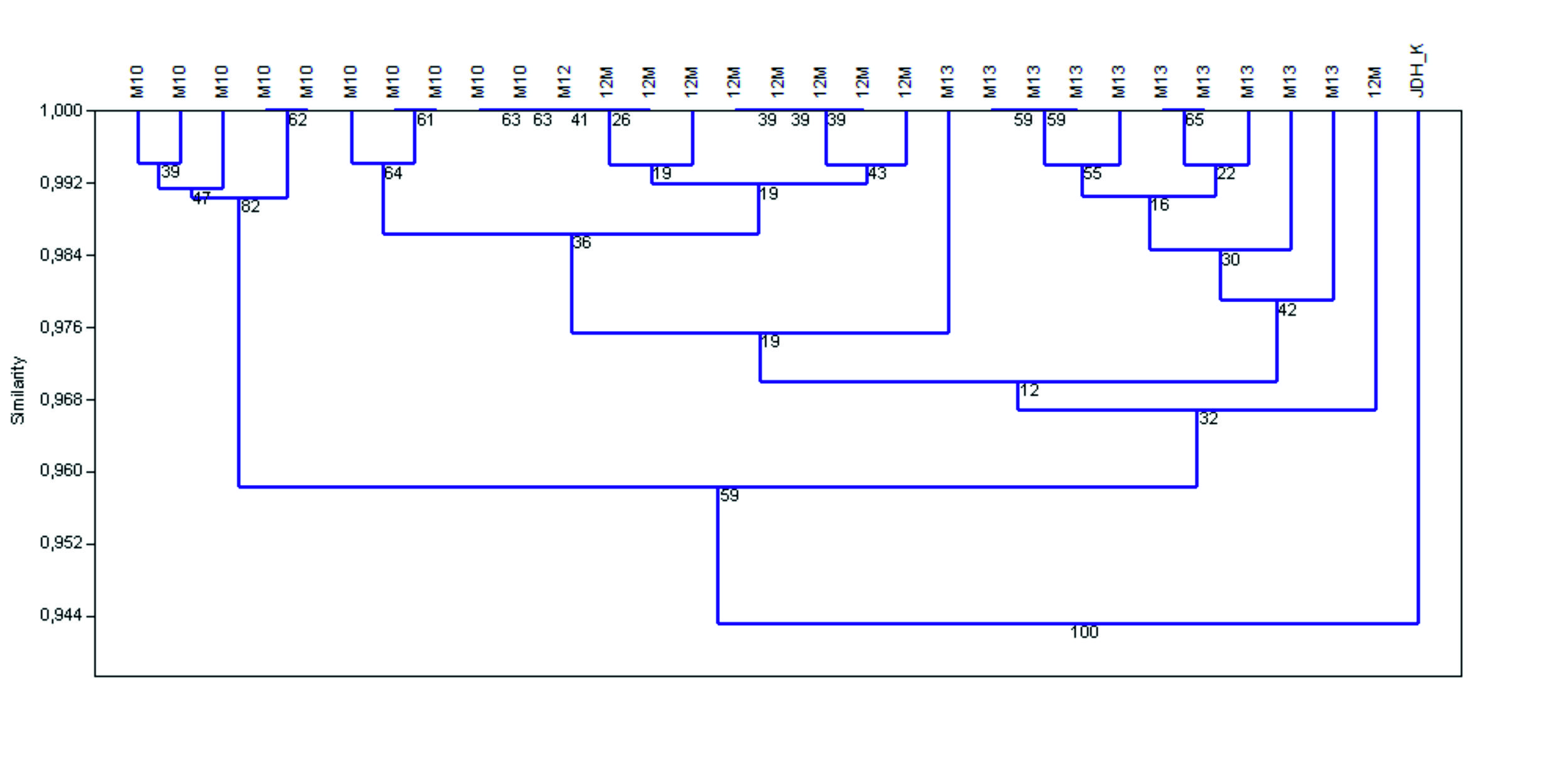

Supplement: Supplementary file 6 — Additional file 6: Figure S5. Agglomeration analysis (UPGMA, Jaccard) conducted on the metAFLPs related to the DNA mutations KpnI/MseI for the M10-M13 trials. The M10 reflects control conditions, whereas the M12 and M13 regenerants derived according to the optimised conditions directed towards the minimum and maximum differences between donor and regenerants. Bootstrap value is indicated on the nodes. [file 40709_2021_138_MOESM6_ESM.jpg]

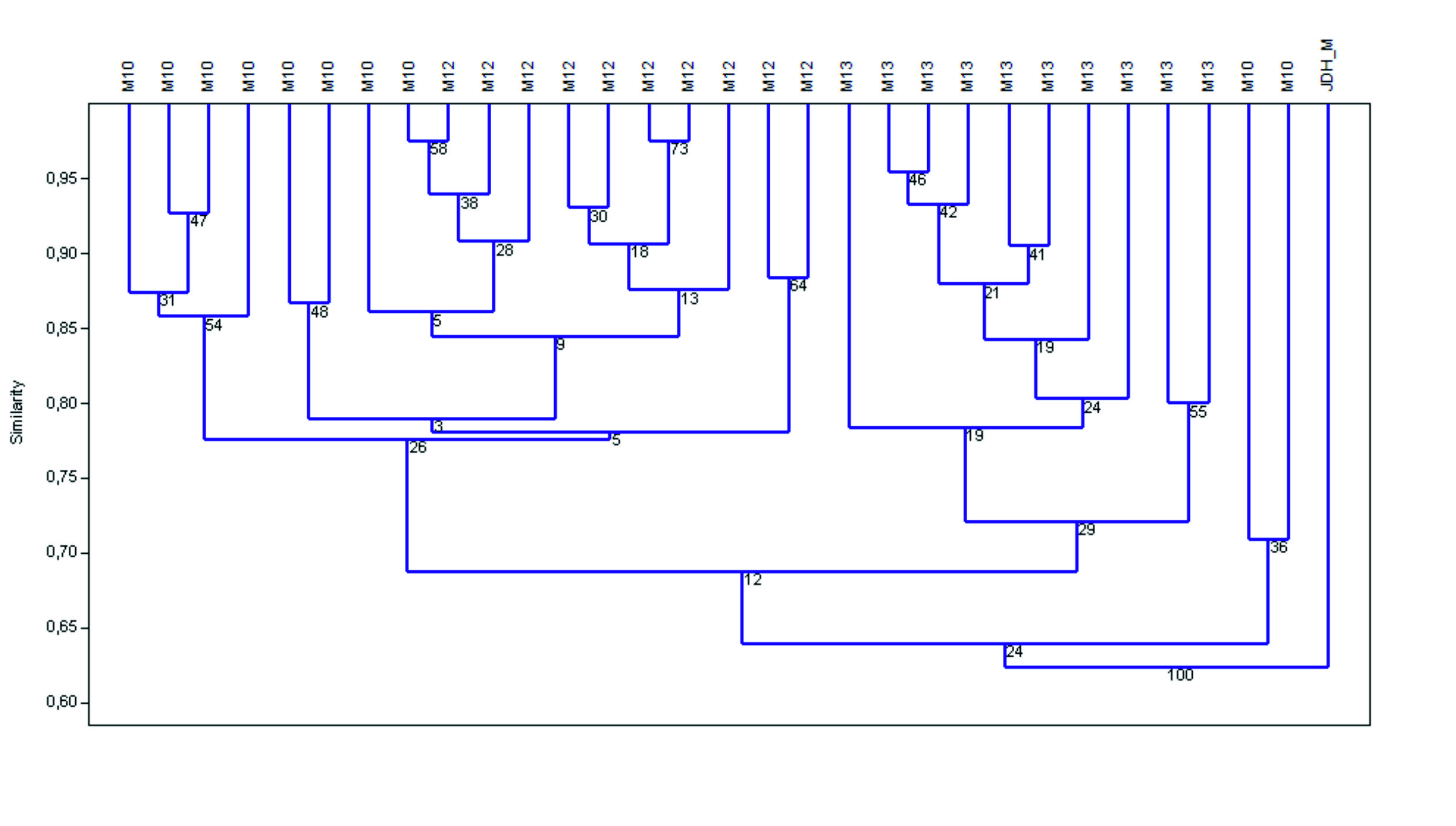

Supplement: Supplementary file 7 — Additional file 7: Figure S6. Agglomeration analysis (UPGMA, Jaccard) conducted on the metAFLPs related to the DNA methylation changes (Acc65I/MseI-KpnI/MseI) for the M10-M13 trials. The M10 reflects control conditions, whereas the M12 and M13 regenerants derived according to the optimised conditions directed towards the minimum and maximum differences between donor and regenerants. Bootstrap value is indicated on the nodes. [file 40709_2021_138_MOESM7_ESM.jpg]
